# Distinct Temporal Structure of Nicotinic ACh Receptor Activation Determines Responses of VTA Neurons to Endogenous ACh and Nicotine

**DOI:** 10.1523/ENEURO.0418-19.2020

**Published:** 2020-08-21

**Authors:** Ekaterina Morozova, Philippe Faure, Boris Gutkin, Christoper Lapish, Alexey Kuznetsov

**Affiliations:** 1 Volen Center for Complex systems, Brandeis University, Waltham, Massachusetts 02453; 2 Neuroscience Paris Seine—Institutde Biologie Paris Seine, INSERM, CNRS, Sorbonne Université, 75013 Paris, France; 3 Group for Neural Theory, LNC2 INSERMU960, Départment d’études cognitives, École Normale Supérieure, Université PSL, 75005 Paris, France; 4 Center for Cognition and Decision Making, Institute for Cognitive Neuroscience, National Research University Higher School of Economics, Moscow 101000, Russia; 5 Department of Psychology, Indiana University-Purdue University at Indianapolis, Indianapolis, Indiana 46204; 6 Department of Mathematical Sciences, Indiana University-Purdue University at Indianapolis, Indianapolis, Indiana 46204; 7 Indiana Alcohol Research Center, Indiana University School of Medicine, Indianapolis, Indiana 46204

**Keywords:** bursting, dopamine neuron, receptor knockout, receptor re-expression, saliency signal, synchrony

## Abstract

The addictive component of tobacco, nicotine, acts via nicotinic acetylcholine receptors (nAChRs). The β2 subunit-containing nAChRs (β2-nAChRs) play a crucial role in the rewarding properties of nicotine and are particularly densely expressed in the mesolimbic dopamine (DA) system. Specifically, nAChRs directly and indirectly affect DA neurons in the ventral tegmental area (VTA). The understanding of ACh and nicotinic regulation of DA neuron activity is incomplete. By computational modeling, we provide mechanisms for several apparently contradictory experimental results. First, systemic knockout of β2-containing nAChRs drastically reduces DA neurons bursting, although the major glutamatergic (Glu) afferents that have been shown to evoke this bursting stay intact. Second, the most intuitive way to rescue this bursting—by re-expressing the nAChRs on VTA DA neurons—fails. Third, nAChR re-expression on VTA GABA neurons rescues bursting in DA neurons and increases their firing rate under the influence of ACh input, whereas nicotinic application results in the opposite changes in firing. Our model shows that, first, without ACh receptors, Glu excitation of VTA DA and GABA neurons remains balanced and GABA inhibition cancels the direct excitation. Second, re-expression of ACh receptors on DA neurons provides an input that impedes membrane repolarization and is ineffective in restoring firing of DA neurons. Third, the distinct responses to ACh and nicotine occur because of distinct temporal patterns of these inputs: pulsatile versus continuous. Altogether, this study highlights how β2-nAChRs influence coactivation of the VTA DA and GABA neurons required for motivation and saliency signals carried by DA neuron activity.

## Significance Statement

Tobacco use remains the worldwide leading cause of preventable mortality. Nicotine, the addictive component of tobacco, exerts its effects through nicotinic acetylcholine receptors. The central dopamine (DA) system, and particularly DA release by neurons contained in ventral tegmental area, is shown to play a central role in developing addictions. Understanding of ACh and nicotinic regulation of DA neuron activity is incomplete, and here we resolve several apparently contradictory experimental results. In particular, we show that distinct responses to ACh and nicotine observed in certain experiments occur because of distinct temporal patterns of these inputs: pulsatile versus continuous. This distinction highlights how motivation and saliency signals carried by DA signaling are hijacked by nicotine and other addictive drugs.

## Introduction

As more than 5 million smokers die every year from the consequences of tobacco use, it remains the worldwide leading cause of preventable mortality (Centers for Disease Control and Prevention, 2017). Underlying neurobiological mechanisms of tobacco dependence have been extensively explored (Taly et al., 2009; Benowitz, 2009, 2010; Changeux, 2010), but remain far from being understood at the neural circuit level. Nicotine, the addictive component of tobacco (Marti et al., 2011), acts via nicotinic acetylcholine receptors (nAChRs; Role and Kandel, 2008). Among the different nAChRs, the β2-containing nAChRs (β2-nAChRs) play a crucial role in positive rewarding properties of nicotine (Picciotto et al., 1998; Durand-de Cuttoli et al., 2018) and is particularly densely expressed in the mesolimbic reward system (Klink et al., 2001).

The mesolimbic reward system, and specifically reward-related dopamine (DA) release throughout the brain, plays a major role in addictive and drug-seeking behaviors (Koob et al., 1998; Everitt and Robbins, 2005; Keiflin and Janak, 2015). DA-releasing neurons have the following two major modes of activity: nearly tonic background firing and bursting. Background firing is responsible for the basal DA levels in the projection areas and is altered in psychiatric disorders from depression to schizophrenia (Grace, 1991; Nestler and Carlezon, 2006). Phasic DA changes, caused by DA neuron burst firing follow external stimuli and are suggested to serve as motivational, saliency, and unexpected reward signals (Schultz, 2002; Redgrave and Gurney, 2006). The bursting mode of the DA neuron is associated with an increase in the occurrence of the behavior that preceded the burst by the mechanism called reinforcement learning (Bayer and Glimcher, 2005; Keiflin and Janak, 2015). DA neurons are contained in the VTA together with GABA neurons. Most drugs of abuse alter DA levels either directly though receptor binding (Lüscher and Ungless, 2006) or indirectly by acting on VTA GABA neurons (Steffensen et al., 2011; Bocklisch et al., 2013). Nicotine influence is very complex to analyze since it enhances DA release by acting on multiple targets on both VTA DA and GABA neurons (Tolu et al., 2013; Faure et al., 2014). Mechanisms of nicotinic influence proposed in previous studies (Mansvelder et al., 2002; Mao et al., 2011) could not explain why, for example, repeated nicotine injections cause excitation of the GABA neurons and yet increase DA release. Therefore, there is a gap in understanding the complex interaction of VTA DA and GABA neurons, which we address through data-based computational modeling.

**Figure 1.**
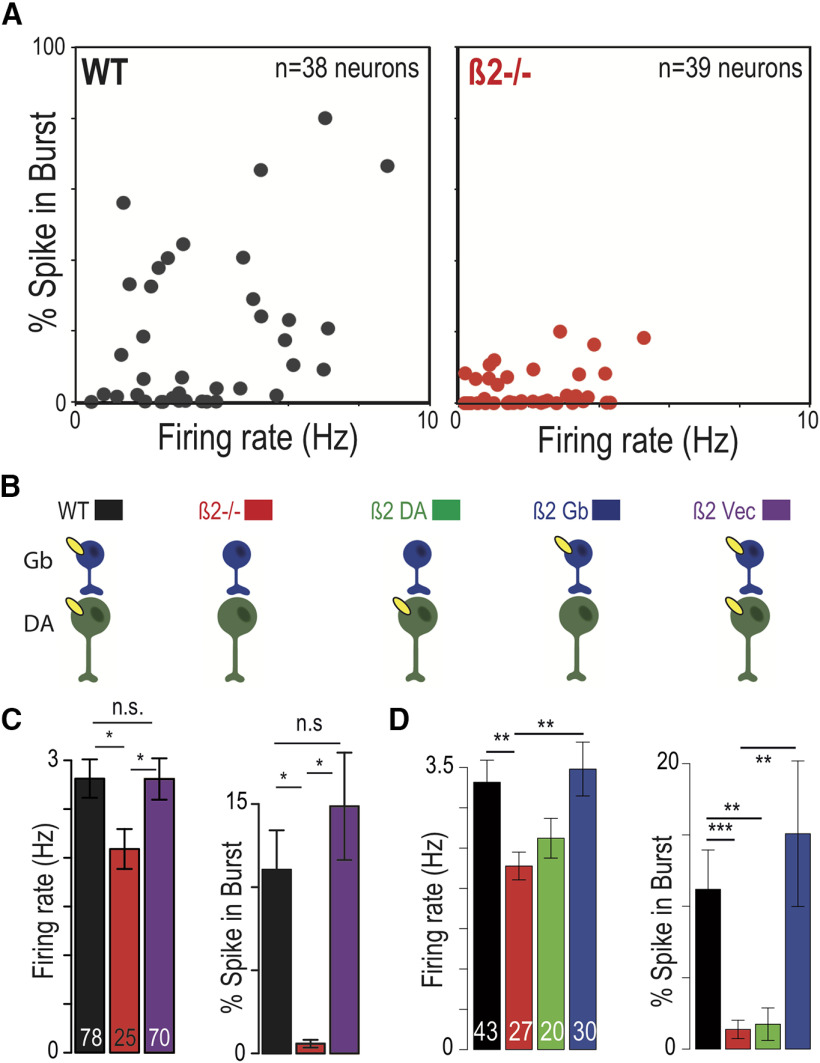
Quantification of firing rate and pattern of the VTA DA neurons in WT mice (black) after systemic deletion of β2-containing nAChRs (red) and their subsequent re-expression on VTA DA (green), on VTA GABA (blue), and on both neurons (purple). ***A***, Mean firing frequency (in Hz) against %SWB for *n* = 38 and *n* = 39 individual cells in WT and β2 KO mice. Systemic deletion of β2-containing nAChR decreases both the DA neuron firing rate and bursting compared with WT (see also ***C*** and ***D***). ***B***, Various KO and re-expression cases that have been used to analyze the role of β2-containing nAChRs in the VTA (see Materials and Methods and ***C***, ***D***). ***C***, Lentiviral re-expression of β2 subunit in the VTA of β2 KO mice using a ubiquitous mouse phosphoglycerate kinase (PGK) promoter (β2-Vec) restores firing rate and bursting (data modified from Naudé et al., 2016). ***D***, Comparison of firing rate and bursting in WT (black), β2 KO mice (β2^−/−^, red), and mice with re-expression of β2 in a specific neuronal population. Cre recombinase-activated lentiviral expression vector was used to drive specific β2*-nAChR re-expression in DA or GABAergic neurons of the VTA of DAT Cre mice (β2 DA, green) and GAD67 Cre mice (β2 Gb, red; Data from Tolu et al., 2013). n.s., *p < 0.05; **p < 0.01; ***p < 0.001 as compared to chance level.

β2-nAChRs are expressed on both VTA DA and GABA neurons. Systemic deletion of the receptors eliminates the bursting mode of DA neurons (Mameli-Engvall et al., 2006; Fig. 1*A*). Such DA neuron bursting is in turn implicated in reinforcement (Bayer and Glimcher, 2005; Keiflin and Janak, 2015). The role of β2-nAChRs on DA or GABA neurons in nicotine reinforcement (i.e., an increase in nicotine consumption) was addressed in previous articles using re-expression of the β2-nAChRs in specific cell populations of the VTA. It has been shown that a global re-expression of the β2-nAChRs in all the VTA (Mameli-Engvall et al., 2006; Naudé et al., 2016) augmented DA neuron firing rate and burstiness compared with β2 knock-out (KO) mice (Fig. 1*B*,*C*, purple vs red). Activation of β2-nAChRs by ACh specifically on the VTA GABA neurons results in increased firing and, importantly, augmented burstiness of DA neurons (Fig. 1*D*, blue vs red). By contrast, the activation of these receptors expressed only on DA neurons by ACh does not produce this effect (Fig. 1*D*, green).

**Figure 2.**
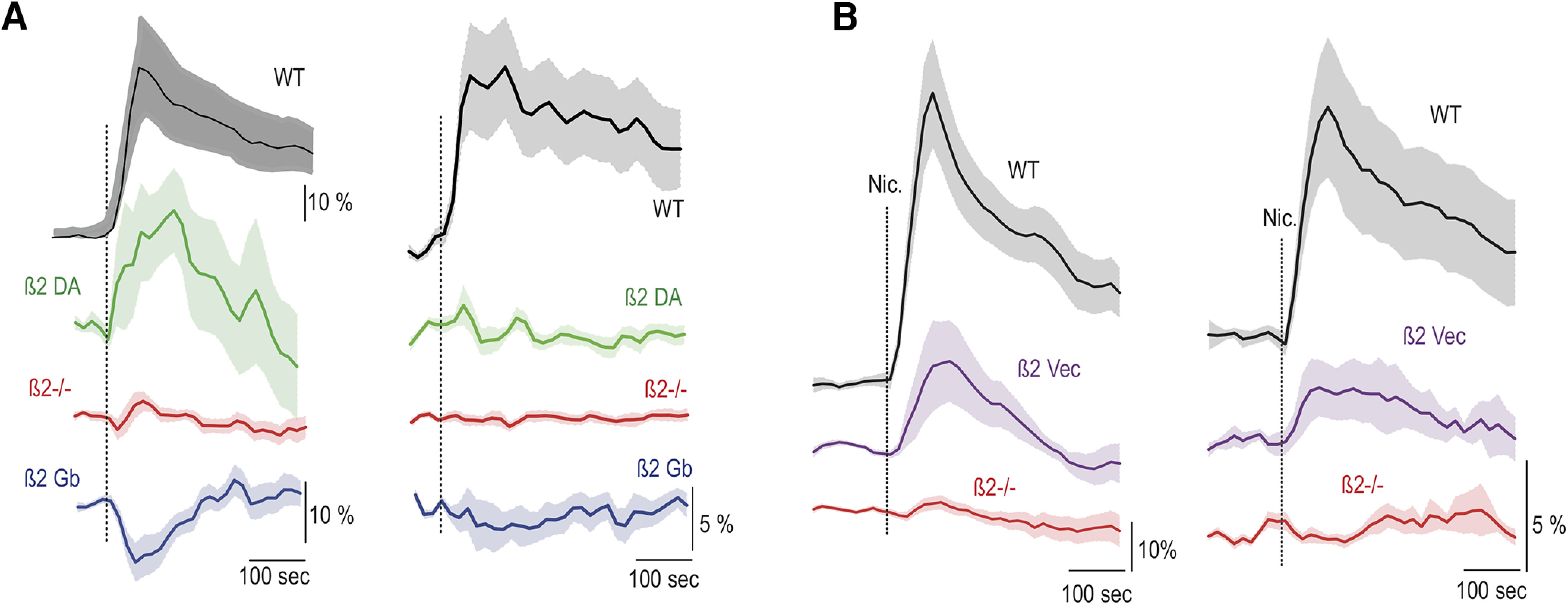
Comparison of the nicotine-elicited modifications of the firing rate (left) and %SWB (right) in VTA DA neurons in in two sets of experiments. ***A***, β2 DA vector (green) and β2 Gb vector (blue) compared with wild-type (black) and β2^−/−^ (red) mice (data modified from Tolu et al., 2013). ***B***, β2-Vec (purple) compared with wild-type (black) and β2^−/−^ (red) mice (data modified from Naudé et al., 2016). Vertical dashed line indicates the nicotine injection.

Adding to the complexity, exposing β2-nAChRs to nicotine produces different effects (Fig. 2, data summarized). When the receptors are re-expressed on the GABA neurons, nicotine increases GABA neuron firing rates and, consecutively, decreases the DA neuron firing rate and bursting (Fig. 2, blue), in contrast to baseline conditions with endogenous ACh input. Nicotine acting through nAChRs expressed only on DA neurons significantly increases DA neuron firing rate, but not bursting (Fig. 2, green; Tolu et al., 2013). Finally, if the receptors are expressed on all VTA neurons (Mameli-Engvall et al., 2006; Tolu et al., 2013; Naudé et al., 2016), nicotine robustly increased the firing rate and bursting of DA neurons (Fig. 2, purple; Naudé et al., 2016). Attempts to explain this bidirectional modulation heuristically, for example by receptor desensitization, have not been successful. We turn to computational modeling to clarify this apparent conundrum. In this article, we analyze the role of local interactions between DA and GABA neurons that mediate the influence of β2-nAChR on the key properties of the DA neuron activity.

By computational modeling, we (1) reconcile the increase of VTA DA cell activity under endogenous ACh with its nicotine-evoked suppression when β2-nAChRs are re-expressed only on the VTA GABA neurons; and (2) show why receptor re-expression restricted to the DA or GABA neurons does not restore nicotine impact on the DA firing to levels seen in wild-type (WT) animals, while re-expression in all VTA neurons does.

## Materials and Methods

### VTA network

**Figure 3.**
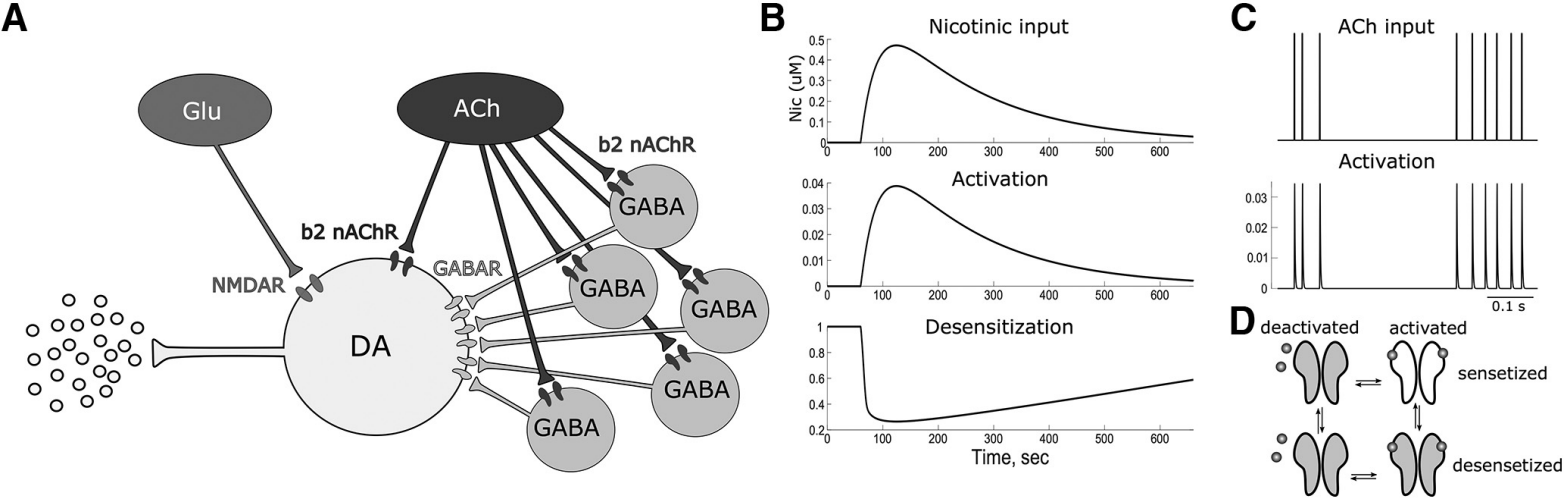
Schematic of the model. ***A***, Afferent inputs and microcircuitry of the VTA. ***B***, Time course of the nicotine concentration and subsequent activation and desensitization of nAChRs. ***C***, Temporal profile of ACh input and subsequent activation of nAChRs. ***D***, State transitions of the nAChRs.

The model network consists of a DA neuron innervated by a population of GABA neurons (Fig. 3*A*). The DA neuron received Glu excitatory input, and both DA and GABA neurons receive cholinergic input via nACh receptors. There are other inputs to this circuit, for example from the nucleus accumbens, but experiments show that re-expression only in the VTA (on all VTA cells) restores all the differences observed in β2^−/−^ knockout (Naudé et al., 2016). Therefore, these afferents are unlikely to be responsible for the observed differences. Thus, other inputs are omitted in the model. The biophysical models of the DA and GABA neurons are conductance-based one-compartment models modified from the study by Morozova et al. (2016a) by adding the following nAChR currents:

$$c_{m}\frac{dv_{\text{DA}}}{dt}=I_{\text{Ca}}+I_{\text{KCa}}+I_{\text{K}}+I_{\text{DR}}+I_{\text{Na}}+I_{\text{sNa}}+I_{\text{leak}}+I_{h}+I_{\text{NMDA}}+I_{\text{AMPA}}+I_{\text{GABA}}+I_{\text{AChDA}}$$

$$c_{m}\frac{dv_{\text{GABA}}}{dt}=I_{K}+I_{\text{Na}}+I_{\text{leak}}+I_{\text{AChGABA}}.$$

For complete description of the model, see Morozova et al. (2016a,b). Briefly, The first eight currents in the DA neuron equation are intrinsic: calcium, calcium-dependent potassium (SK), subthreshold potassium, spike-producing potassium-delayed rectifier and sodium, subthreshold sodium, leak, and hyperpolarization-activated cationic current (*h*). The rest of the currents are synaptic: NMDA, AMPA, GABA_A_, and nACh receptors. The GABA neuron includes only spike-producing sodium and potassium, the leak intrinsic currents, and the nACh receptor current. Gating variables for all currents are modeled in the standard form $\frac{dX}{dt}=\frac{X_{\text{inf}}\left(V\right)-X}{\tau_{X}}$, where *X* denotes a specific gating variable, $X_{\text{inf}}\left(V\right)$ is the function of its steady-state activation, and $\tau_{X}$ is its time constant. The model has been calibrated using *in vitro* and *in vivo* data in previous publications (Morozova et al., 2016a,b). We list the values for all parameters in Extended Data Table 1-1 and refer the reader to the above publications for detailed explanations.

### Cholinergic currents

We modeled only the nAChR currents on VTA neurons because other currents affected by cholinergic inputs are not changed in the KO conditions, and, thus, cannot cause the observed differences. The nAChR currents on DA and GABA neurons are given by the following expressions respectively: $I_{\text{AChDA}}=g_{\text{AChDA}}(E_{\text{ACh}}-v_{\text{DA}})$ and $I_{\text{AChGABA}}=g_{\text{AChGABA}}(E_{\text{ACh}}-v_{\text{GABA}})$. Here, $E_{\text{ACh}}=0$. The model of nAChR activation and desensitization was adapted from the study by Graupner et al. (2013). Note that the approach has been also validated and used to partially explain data in the study by Tolu et al. (2013). The model for the nAChR-mediated currents has four different states of the nAChR: deactivated/sensitized (also resting or responsive state); activated/sensitized; activated/desensitized; and deactivated/desensitized state (Graupner et al., 2013). The mean total activation level of nAChRs is a product of the fraction of receptors in the activated state ${\text{Ach}}_{\text{act}}$ and the fraction of receptors in the sensitized state $\left(1-{\text{ACh}}_{\text{des}}\right)$; thus, conductance of the cholinergic current is given by $g_{\text{Ach}}={\bar{g}}_{\text{Ach}}\cdot {\text{Ach}}_{\text{act}}\cdot (1-{\text{Ach}}_{\text{des}})$. The time course of the activation and desensitization variables is given by $\frac{{\text{dACh}}_{\text{act}}}{dt}=\frac{{\text{ACh}}_{\text{act¥}}-{\text{ACh}}_{\text{act}}}{\tau_{{\text{ACh}}_{\text{act}}}}$ and $\frac{{\text{dACh}}_{\text{des}}}{dt}=\frac{{\text{ACh}}_{\text{des¥}}-{\text{ACh}}_{\text{des}}}{\tau_{{\text{ACh}}_{\text{des}}}}$ respectively. $\tau_{{\text{ACh}}_{\text{act}}}=5\text{ms}$ is an activation time constant, $\tau_{{\text{ACh}}_{\text{des}}}=500+6\cdot 10^{5}\cdot \frac{1}{1+{\left(\frac{{\text{inp}}_{\text{Nic}}}{K_{\tau }}\right)}^{3}}\text{ms}$ is a nicotine concentration-dependent desensitization time constant. Steady states ${\text{Ach}}_{\text{act}\infty }$ and ${\text{Ach}}_{\text{des}\infty }$ are given by Hills equations of the form ${\text{Ach}}_{\text{act}}=\frac{1}{\left(1+{\left(\frac{{\text{EC}}_{50}}{{\text{inp}}_{\text{Ach}}+w\cdot {\text{inp}}_{\text{Nic}}}\right)}^{1.05}\right)}$ and ${\text{Ach}}_{\text{des}}=\frac{1}{\left(1+{\left(\frac{{\text{IC}}_{\text{50}}}{{\text{inp}}_{\text{Nic}}}\right)}^{0.5}\right)}$. Here, EC_50_ and IC_50_ values are the half-maximal concentrations of nAChR activation and desensitization respectively. w is the potency of nicotine to evoke a response. The parameters for the cholinergic currents were calibrated in previous publications (Graupner et al., 2013; Tolu et al., 2013). We refer the reader to these publications for detailed explanations and list the values in Extended Data Table 1-1, except for the parameters recalibrated in this study that are listed in Tables 1 and 2.

**Table 1:** Model parameters (see **Extended Data Table 1-1 for full list)**

Parameter	Description	DA neuron	GABA neuron
${\text{Ainp}}_{\text{Ach}}$	Amplitude of Ach input	5 μM	10 μM
${\text{Ainp}}_{\text{Nic}}$	Amplitude of Nic input	0.5 μM	0.5 μM
w	Potency of Nic to evoke response	3	3
$g_{\text{GABA}}$	GABAR conductance	2.5 mS/cm^2^	
$g_{\text{NMDA}}$	NMDAR conductance	4 mS/cm^2^	0 mS/cm^2^
$g_{l}$	Leak conductance	0.03 mS/cm^2^	0.05 + 0.05(rnd-0.5) mS/cm^2^

**Table 2:** ACh receptor maximal conductance used to reproduce different KO re-expression cases

Parameter	KO	Re-expression on DA	Re-expression on GABA	Re-expression on both, WT case
${\bar{g}}_{\text{AChDA}}$	0	5 mS/cm^2^	0	10 mS/cm^2^
${\bar{g}}_{\text{AChGABA}}$	0	0	4 mS/cm^2^	1.5 mS/cm^2^

Nicotine has relatively slow pharmacodynamics in the brain; hence, the application of nicotine was modeled as a slow increase in ${\text{inp}}_{\text{nic}}$ causing slow activation of the nAChRs followed by yet slower desensitization. On the other hand, endogenous cholinergic input to the VTA was temporally structured as a spike train with a bimodal distribution to achieve a bursty firing pattern. The majority of neurons in laterodorsal tegmental nucleus (LTDg) and pedunculopontine tegmental nucleus are generally slow, maintaining individual firing rate averages between 2 and 5 Hz (Kayama et al., 1992; Sakai, 2012). Putative cholinergic neurons transiently increase their firing rate, reaching 10 Hz in response to sensory stimuli (Koyama et al., 1994). It has been shown that LTDg neurons are essential for DA neuron burst firing. However, microiontophoretically applied acetylcholine onto identified DA neurons, while inactivating the LDTg failed to induce burst firing in DA neurons (Lodge and Grace, 2006). This likely suggests that the temporal structure of cholinergic input onto VTA neurons is crucial for DA neuron burst firing. The number of these afferent neurons converging on each VTA neuron is not known. We assume this number to be in the range of 20–60, which has been suggested for other subcortical afferents. Multiplying the number of afferent neurons by the rate, under the assumption that the projecting neurons are asynchronous, gives us the average ACh afferent input frequencies of 60–180 Hz. If ACh afferents are synchronized, these rates will be lower, whereas the amplitude of this input will be greater. Thus, we position the peaks of the frequency distributions for ACh input at 3.3 and 30 Hz (i.e., interspike intervals of 300 and 30 ms). The ACh input spike train was generated by drawing the interspike intervals from the two normal distributions with SD of 7 Hz and their relative contributions of 0.7 and 0.3 for 30 and 3.3 Hz, respectively (Fig. 3*B*).

Assuming that the intravenous nicotine concentration slowly builds up at the site of the receptor, it increases and then decays exponentially in the model with a rise time constant of 0.5 min and a decay time constant of 3 min (Fig. 3*C*), as follows:

$${\text{inp}}_{\text{Nic}}={\text{Ainp}}_{\text{Nic}}(e^{-\frac{t}{\tau_{\text{rise}}}}-e^{-\frac{t}{\tau_{\text{decay}}}}).$$

The amplitude of the nicotinic input ${\text{Ainp}}_{\text{nic}}$ is assumed to be lower by a factor of 10 than that of ACh (Table 1) because, by contrast to nicotine, ACh is released locally at the synapse. The concentrations match those projected from experiments (Garzón et al., 1999). The model is coded in MATLAB/c++, and the code is available on ModeDB database (https://senselab.med.yale.edu/modeldb/ShowModel?model=266419&file=/modelDB_NicandACh/mainNicandAChDAmodel.m#tabs-1).

### DA neuron firing pattern quantification

According to the classical definition (Grace and Bunney, 1984), bursts were identified as discrete events consisting of a sequence of spikes with burst onset defined by two consecutive spikes within an interval of <80 ms, and burst termination defined by an interspike interval of >160 ms. To quantify bursting, we used the percentage of spikes within burst (%SWB), calculated as the number of spikes within bursts divided by the total number of spikes. In our simulations, the majority of bursts was composed of doublets; this is consistent with data (Grace and Bunney, 1984; Mameli-Engvall et al., 2006; Exley et al., 2011). In experiments (Mameli-Engvall et al., 2006), the mean number of spikes in WT mice in a burst was 3.1 ± 0.52. In our simulations, the average length was 2.7 ± 0.25 spikes/burst, which is within the range observed in the experiments.

## Results

With our model, we give an account in silico for the ACh-induced and nicotine-induced modulations observed in the experiments. We explain and provide mechanisms for several apparently contradictory experimental results. First, systemic KO of β2-containing nAChRs drastically reduces DA neuron bursting, although the major Glu afferents that have been shown to evoke this bursting stay intact. Second, the most intuitive way to rescue this bursting by re-expressing the nAChRs on VTA DA neurons fails. Third, nAChR re-expression on VTA GABA neurons rescues DA neuron bursting under the influence of ACh input, but nicotinic application results in the opposite changes in VTA DA neuron firing.

### Balanced excitatory and inhibitory inputs to DA neurons support their tonic firing in the β2-nAChR knock-out conditions

**Figure 4.**
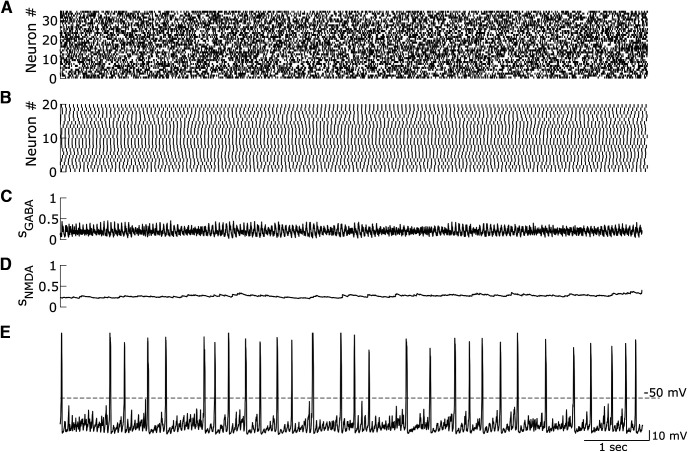
The rate and regularity of the DA neuron firing receiving asynchronous synaptic Glu and GABA inputs. ***A***, Glu raster. ***B***, GABA raster. ***C***, Activation of the GABAR on the DA neuron. ***D***, Activation of the NMDAR on the DA neuron. ***E***, The voltage of the DA neuron. Note the high regularity of DA neuron firing.

In animals lacking β2-containing nAChRs, VTA DA neurons were shown to fire at lower frequencies than the controls and to display practically no bursting *in vivo* (Figs. 1, 2). Burst firing of VTA DA neurons has been previously, which, at least in part, is attributed to the activation of their Glu synaptic inputs (Morikawa et al., 2003; Blythe et al., 2007; Deister et al., 2009). This leads to a question about why the knockout of β2-containing nAChRs abolished bursting in DA neurons if their Glu inputs remain intact. Considering that some excitatory inputs to the VTA DA neurons are tonically active (e.g., STN; Wilson et al., 2004), we modeled the Glu inputs as Poisson-distributed spike trains, which together produce near-tonic activation of NMDA receptors on DA neurons. Simultaneously, the DA neurons receive inhibitory inputs (Paladini and Tepper, 1999; Kaufling et al., 2010), which in the model are provided by population activity of VTA GABA neurons. While Glu inputs alone through NMDA receptor activation can cause increases in DA neuron firing and bursting, coactivation of GABA receptors can balance the excitation and re-establish low-frequency near-tonic firing (Lobb et al., 2010). This behavior has been reproduced in previous modeling studies (Morozova et al., 2016a). Accounting for the low-rate tonic firing activity of the DA neuron required the GABA receptor (GABAR) and NMDA receptor (NMDAR) conductances in the model to be set to produce a balance in the GABA and Glu inputs to the DA neuron (Fig. 4). Thus, the model implies that in the β2-nAChR KO conditions the excitatory and inhibitory inputs to VTA DA neurons remain balanced and preserve tonic activity of the neurons.

### Intrinsic excitability properties of the DA neuron limit effects of direct excitation via β2-containing nAChRs

The most intuitive explanation of the bursting of low-DA neurons in β2-nAChR KO mice would be the lack of direct excitation through these receptors on DA neurons. However, targeted re-expression of the receptors on DA neurons (b 2DA-VEC) does not alleviate low bursting (Fig. 1, green). The firing rate also remains much lower than in the WT conditions. The main effect of this re-expression is that nicotine application produces a robust increase in the firing rate of DA neurons (Fig. 2, green), similar to that in WT conditions. However, bursting is not increased during the influence of nicotine (Fig. 2, green), by contrast to that in the WT mice.

**Figure 5.**
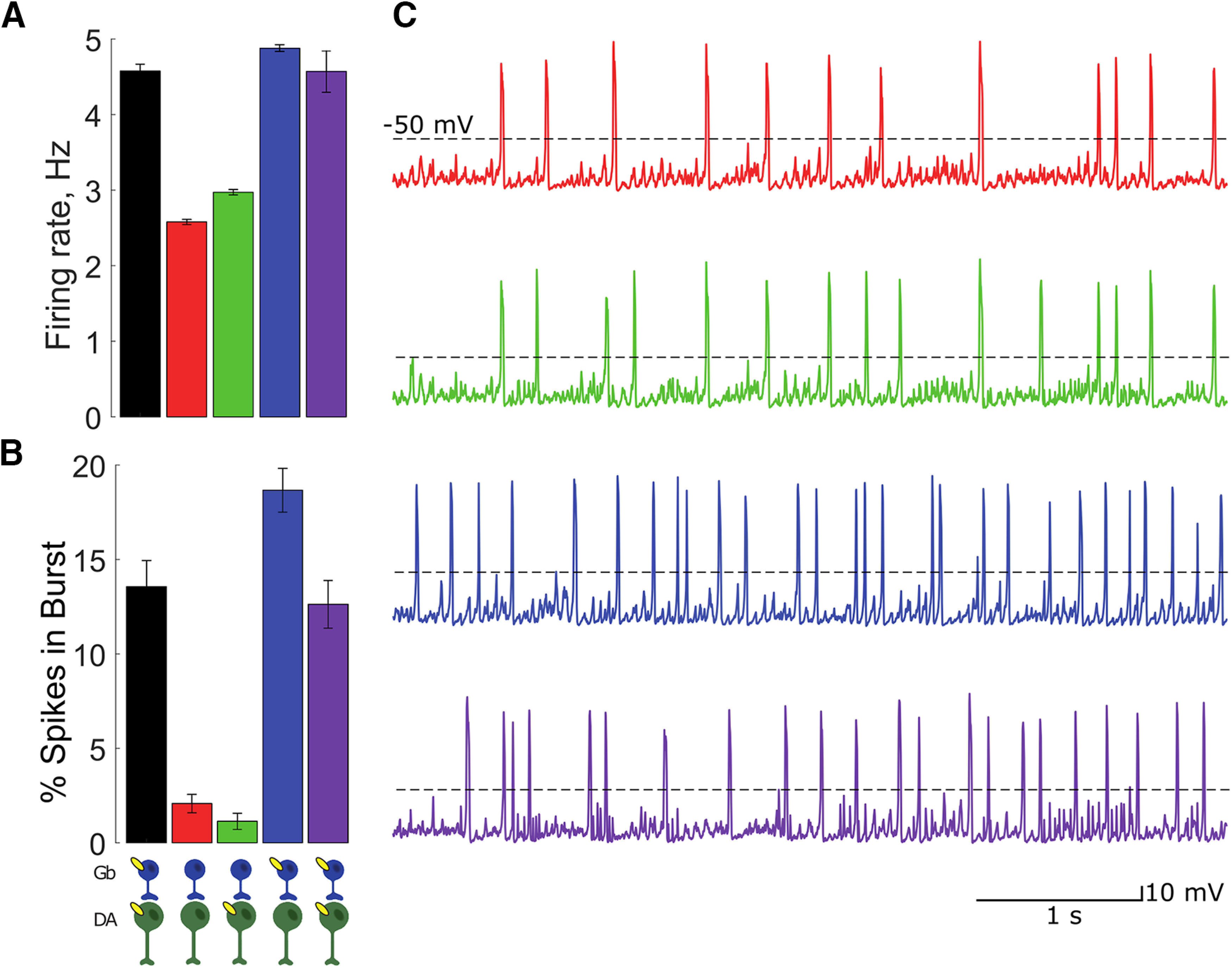
Quantification of the spontaneous firing of simulated DA neurons. ***A***, Bar plot representation of the mean basal firing rate, represented as the mean ± SEM, WT case (black), KO (red), β2-containing nAChRs on DA neurons (green), the nAChRs on GABA neurons (blue), and the nAChRs on DA and GABA neurons (purple). ***B***, and mean %SWB. ***B***, Bar plot representation of the mean %SWB, represented as the mean ± SEM, KO (red), the nAChRs on DA neurons (green), the nAChRs on GABA neurons (blue), and the nAChRs on DA and GABA neurons (purple). ***C***, Example voltage traces of simulated DA neurons under four different conditions, indicated in ***A*** and ***B***. Colors of the voltage traces match the colors of the bars. Inclusion of nAChR-mediated ACh current to GABA neurons significantly increased DA neuron firing and bursting in a manner similar to the experiment (Tolu et al., 2013). See Extended Data Figure 6-1 for parametric analysis.

Conductance of the nAChR current was set to a low value (${\bar{g}}_{\text{AchDA}}=5{\text{mS/cm}}^{\text{2}}$) to reproduce the lack of significant change in the firing rate of the β2DA-VEC (experiment: Fig. 1, green; model: Fig. 5, green) compared with the KO case (experiment: Fig. 1, red; model: Fig. 5, red). For these low conductance values, bursting was also not altered by the direct ACh inputs on the DA neurons. The choice of the conductance value was based on our parametric analysis of its influence on the DA neuron firing rate and bursting included in Extended Data Fig. 5-1. Thus, the model reproduces experimentally observed invariance of the VTA DA cell activity under the direct nAChR-mediated cholinergic input to the DA neurons.

**Figure 6.**
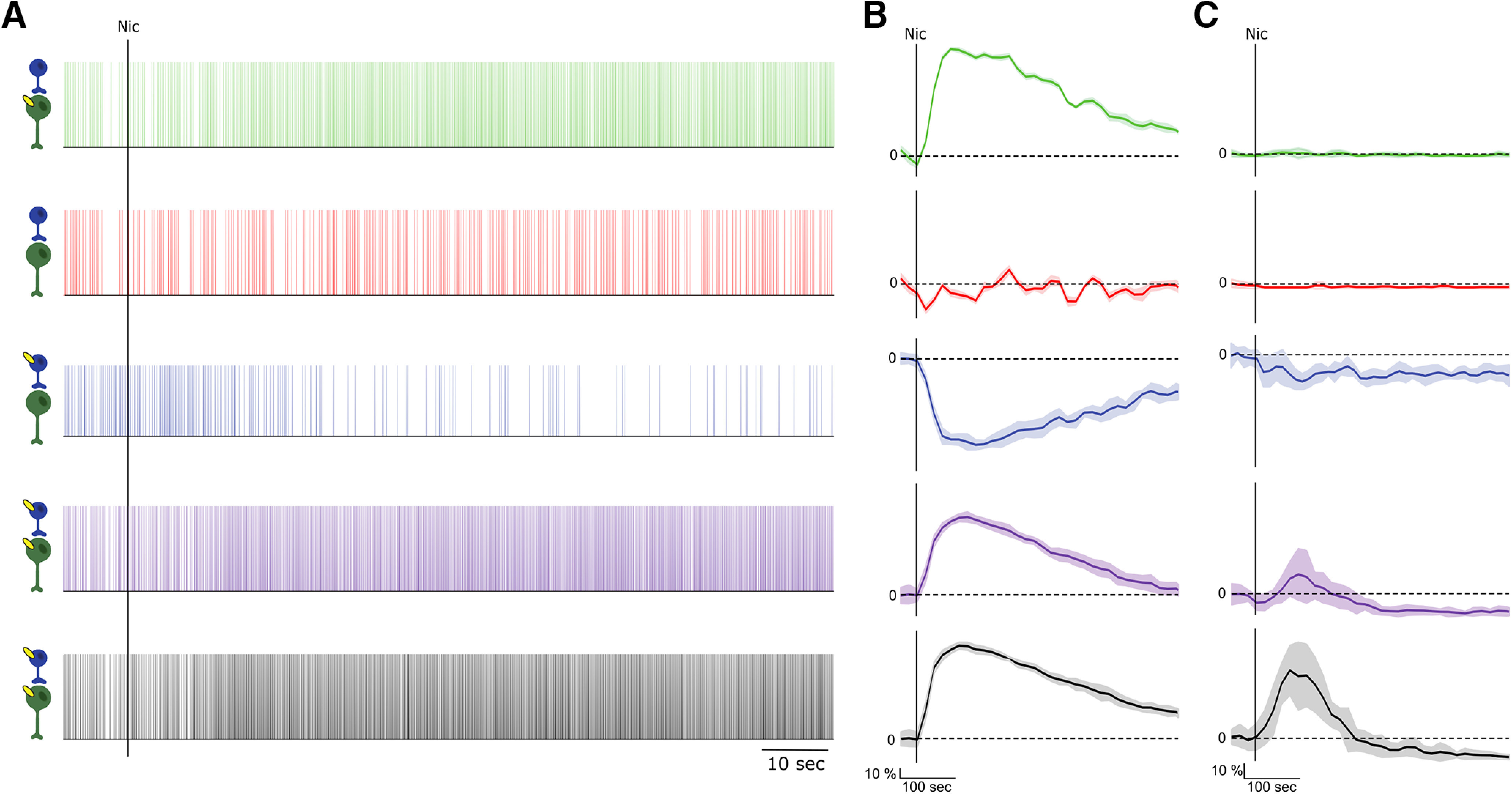
Nicotine-elicited changes in firing rate and burstiness of simulated DA neuron. ***A***, Raster plots of DA neuron firing in response to nicotine, KO (red), β2-containing nAChRs on DA neurons (green), the nAChRs on GABA neurons (blue), the nAChRs on DA and GABA neurons (purple), and WT case (black). ***B***, ***C***, Nicotine-elicited changes in firing rate (***B***) and %SWB (***C***) of simulated DA neurons in response to nicotine. The vertical black line shows the onset of the nicotinic input. There is no change in DA neurons firing or bursting in response to nicotine if both DA and GABA neurons lack the receptors (red). Nicotine increases DA neuron firing if β2-nAChR is added only to the DA neuron (green). Oppositely, it decreases DA neuron firing and bursting if β2-nAChR is added only to GABA neurons (blue). Interestingly, nicotine increases DA neuron firing and bursting if β2-nAChRs are added to both neurons (purple). Nicotine elicits an even greater response in the WT-like case, because of the nicotine-elicited increase in the frequency of Glu inputs to the DA neurons (black). See Extended Data Figure 6-1 for parametric analysis.

10.1523/ENEURO.0418-19.2020.f6-1 Figure 6-1 Parametric analysis of DA neuron responses to ACh and nicotinic inputs for different maximal conductances of nAChR current (mimicking different levels of expression of nAChRs) on GABA neurons. The range of low nAChR conductances on GABA neurons shows good correspondence with the experimental data. Download Figure 6-1, TIF file.

10.1523/ENEURO.0418-19.2020.tab1-1 Table 1-1 Model parameters. Download Table 1-1, DOCX file.

10.1523/ENEURO.0418-19.2020.f5-1 Figure 5-1 Parametric analysis of DA neuron responses to ACh and nicotinic inputs for different maximal conductances of nAChR current (mimicking different levels of expression of nAChRs) on DA neurons. The range of low nAChR conductances shows a good correspondence with the experimental data. Download Figure 5-1, TIF file.

In addition to the cholinergic input, we were able to account for the effects of the nicotine application. As in the experiments, the firing rate, but not the bursting of the DA neuron, substantially increases with activation of the β2 receptors for ∼300 s by elevated nicotine concentration (Fig. 6, green). The increase is significant, by contrast to that produced by ACh alone. This simply reflects a greater increase in the nAChR-mediated current by the combination of nicotine and ACh. Desensitization of the receptors will eventually decrease their current, but at a much longer time scale of 1 min.

The explanation for the lack of increase in burstiness during nicotine exposure is that bursting in DA neurons strongly relies on the activation of the NMDA receptor (Overton and Clark, 1992; Chergui et al., 1993; Deister et al., 2009) because of its voltage dependence (magnesium block; Deister et al., 2009; Ha and Kuznetsov, 2013). By contrast, tonic activation of linear current mediated by the nicotinic receptors is not effective in eliciting bursting in DA neurons. In fact, we can think about the nAChR-mediated current as being similar to a slow-varying AMPA input, since both are not voltage dependent, as opposed to the voltage-rectifying NMDA–synaptic currents. Therefore, even strong continuous increase in the nAChR-mediated current on the VTA DA neuron does not increase its bursting.

### Distinct temporal profiles of AChR activation explains opposite effects of endogenous ACh and nicotine on VTA DA neuron firing

Counterintuitively, when β2-containing nAChRs are re-expressed only on VTA GABA neurons, endogenous ACh input and exogenous nicotinic application results in opposite changes in firing of VTA DA neurons. In particular, the firing rate and especially the bursting of VTA DA neurons are sharply increased after the re-expression of β2-nAChRs on VTA GABA neurons compared with the KO conditions (experiment: Fig. 1; model: Fig. 5, blue vs red). By contrast, the additional agonist effect on these receptors by nicotine causes a decrease in the firing and bursting of the DA neuron (experiment: Fig. 2, blue; model: Fig. 6, blue). While the decrease in the firing caused by nicotine can be intuitively explained by a greater activation of the GABA neurons, which in turn inhibits the DA neurons, the boost in activity of DA neurons produced by ACh is harder to understand. We show that our model is capable of accounting for these effects in a mechanistic manner.

**Figure 7.**
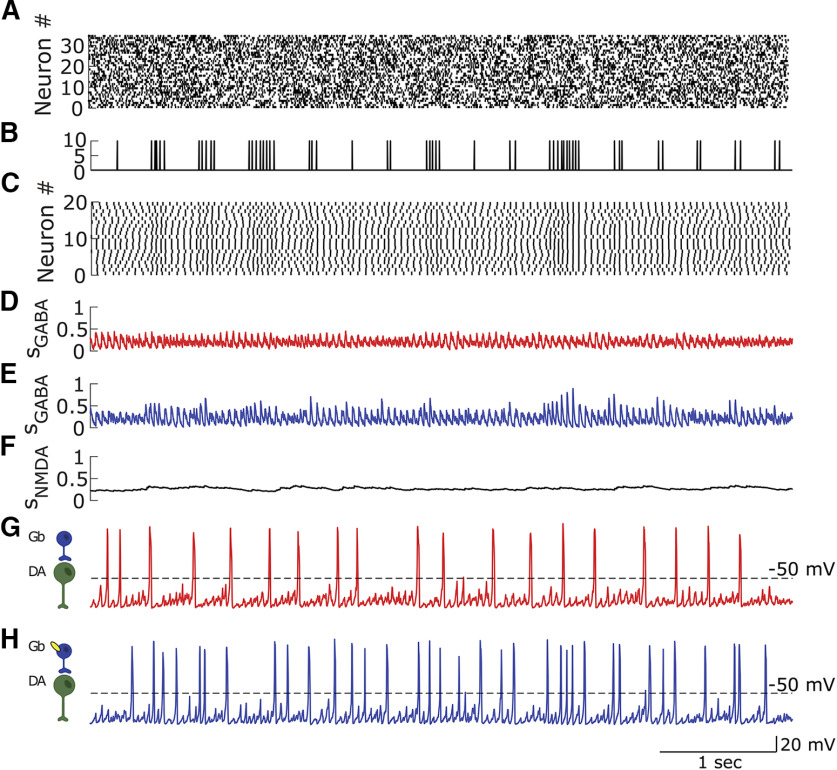
Bursty pulsatile cholinergic input transiently synchronizes GABA neurons. Synchronous GABA input evokes additional DA spikes and increases DA neuron burstiness. ***A***, Raster of Glu neurons. ***B***, ACh input. ***C***, Raster of VTA GABA neurons. ***D***, ***E***, The cumulative activation variable of the GABAR current on DA neurons without (red) and with (blue) ACh input. ***F***, The activation variable of the NMDAR current on the DA neuron. Note the lack of significant variations. ***G***, ***H***, Voltages of the DA neurons in the cases where they receive GABAR activation from ***D*** or ***E***, respectively. Note that there is a greater number of spikes grouped in bursts when the nAChR is added to the GABA neurons (blue).

As before, cholinergic input arrving at the GABA neurons was modeled by a spike train reproducing the burstiness of the afferents (see Materials and Methods). Following the afferent convergence principle, we assumed that a subpopulation of VTA GABA neurons receives common endogenous cholinergic input that activates the β2-nAChRs on these neurons. Previously, we have shown that such common input can synchronize the GABA neurons and functionally invert their inhibitory influence on DA neurons (Morozova et al., 2016a). We showed that a synchronous pulsatile GABA input is able to significantly increase the firing rate and burstiness of the DA neurons via dynamic reduction of the Ca^2+^-dependent K^+^ current. This mechanism works here for the influence of ACh inputs through the GABA neurons onto the DA neurons: because of fast, transient activation of nAChRs, cholinergic pulses act as synchronizing inputs to GABA neurons (Fig. 7). The synchronized GABA synaptic input onto the DA neuron acts in turn to increase its firing rate and burstiness. Thus, experimentally observed increases in firing and bursting of the DA neurons that follow nAChRs re-expression on VTA GABA neurons could be mechanistically explained by changing the level of synchronization in the population of the GABA neurons (Morozova et al., 2016a).

By contrast to the pulsatile activation of nAChRs by ACh afferents, nicotine activates nAChR on GABA neurons tonically and then desensitizes them. This produces an increase in GABA neuron firing rate without synchronizing the neurons (data not shown). This in turn leads to an increase in the tonic component of GABA synaptic activation on DA neurons. Tonic GABA activation on DA neurons leads to a decrease in their firing, which allows us to reproduce the inhibitory influence of nicotine through GABA neurons (Fig. 6, blue, Extended Data Fig. 6-1, parameter dependence).

### Coactivation of β2-nAChRs on VTA DA and GABA neurons is required for both ACh and nicotine to boost DA neuron bursting

Under the ACh input, re-expression of β2-containing nAChRs on all VTA neurons elevated the firing rate and burstiness of DA neurons to the levels displayed in the WT animals (Fig. 1*B*). We have reproduced this modulation in the model (Fig. 5, purple). However, this required a modification of the nAChR conductances compared with the above re-expression cases (Table 2). The model shows that the ACh-induced and nicotine-induced modulations strongly depend on the balance between the expression of these receptors on DA and GABA neurons. For example, if we combine the conductances at the values used in the two single re-expression cases, both firing rate and bursting will be very high under the ACh input, but will decrease with nicotinic application, which is opposite to experiments. Therefore, nAChR conductance was reduced by a factor of 2.6 on the GABA neurons and increased by a factor of 2 on the DA neuron. Thus, model calibration predicts that the elevated firing rate and burstiness caused by coexpression of nAChRs on both the GABA and the DA neurons, although attaining very similar values, rely in a very different mechanism: the firing rate is increased because of a direct ACh-mediated excitation of DA neurons, whereas GABA neurons influence augments bursting.

Nicotine application after re-expression of β2-containing nAChRs on both VTA DA and GABA neurons produced increases in both the firing rate and bursting of DA neurons in experiments (Fig. 2, purple). To model this case, nicotinic input was applied to both DA and GABA neurons. The resulting DA neuron firing rate is produced by a competition between nicotine-evoked direct excitation and GABA-mediated inhibition. We used the same parameters as in the case of ACh influence on both neurons above. The overall increase in DA neuron firing rate was achieved because of a stronger direct excitation of DA neurons than their inhibition through GABA neurons by nicotine (Fig. 6, purple). However, as it follows from the case of nAChRs re-expression only on DA neurons, this pathway cannot be implicated in increasing bursting of the DA neurons. The mechanism for increasing bursting combines the increase in direct excitation and GABA neuron-mediated inhibition of DA neurons. We have shown that this combination effectively increases DA neuron bursting as spikes within bursts are more resistant to inhibition than those without (Morozova et al., 2016a; Di Volo et al., 2019). Thus, increased firing rate and bursting of DA neurons induced by nicotine application through β2-containing nAChRs suggests the convergence of both direct excitation and GABA-mediated inhibition, with the latter having a weaker influence.

### A combination of nicotinic activation of β2-containing nAChRs on VTA neurons and Glu projections underlies modulation of DA neuron firing in wild-type mice

Re-expression of β2-containing nAChRs on all VTA neurons supposedly restores the influence of ACh and nicotine within, but not outside of, the VTA. Therefore, some distinctions from firing rates and patterns of DA neurons in WT mice may remain. In the absence of nicotine, the firing rate and bursting of DA neurons in WT mice are not statistically different from that in the whole VTA re-expression case (Fig. 1, black). However, nicotine application increases bursting and firing rate to greater levels in WT mice compared with the whole VTA re-expression case (Fig. 2, black).

In simulations, we use the same conductances of the nAChRs on both DA and GABA neurons as in the double re-expression case above. To account for nicotinic modulation in other brain regions as the nAChRs are preserved throughout the brain, we increase the average frequency of Glu afferents from 50 to 60 Hz for the duration of the nicotine injection. The increase caused greater DA neuron firing rates and bursting compared with double re-expression case (Fig. 6, compare black, purple) during nicotinic excitation of the receptors, matching the experimental results. Therefore, a combination of modulations produced by nicotine inside and outside of the VTA allows us to reproduce the impact of nicotine administration on the firing properties of the VTA DA neurons.

## Discussion

The above results provide essential details of how nicotine impacts the drug reinforcement-related circuitry in the VTA. The amplified phasic DA output of the circuit requires concerted activation of β2-nAChRs on both DA and GABA neurons in the VTA (Tolu et al., 2013). Our modeling suggests that direct excitation of DA neurons by endogenous ACh via nAChRs is not enough to increase bursting. In fact, coactivation of GABA neurons via the same receptor activation does not exert the suggested inhibitory influence but is necessary for bursting. The mechanism that allows us to reproduce the experimental results is the modulation of synchrony levels in the VTA GABA neural population. Synchronization among GABA neurons produces pulsatile hyperpolarizing input to the DA neurons that increases their firing and bursting (Morozova et al., 2016a). A pulsatile GABA input dynamically reduces long-lasting intrinsic inhibition (Ca^2+^-dependent K^+^ currents), and the pulsatile pattern of the external inhibition allows the DA neurons to escape it and fire spikes between the pulses. This promotes the occurrence of extra DA neuron spikes via phasic disinhibition. On the contrary, a desynchronized GABA population provides a nearly constant level of inhibition to DA neurons, which suppresses their firing. The difference in synchrony can be produced by ACh input versus nicotinic injection. Because of fast transient activation of β2-nAChRs, ACh pulses can act as synchronizing inputs to these GABA neurons. By contrast, nicotine persistently activates nAChRs, causing an increase in the frequency without synchronizing the GABA population. This study highlights the role of GABA interneurons and, in particular, the temporal pattern of their activity in the regulation of the bursting of DA neurons.

Our modeling has reconciled apparent controversies of complex cholinergic and nicotinic modulation of the VTA DA neuron firing rate and pattern. First, systemic KO of β2-nAChRs drastically reduces bursting of DA neurons. DA neuron bursting have been shown to depend on the Glu inputs (Morikawa et al., 2003; Blythe et al., 2007; Deister et al., 2009) and has a major behavioral function as an unexpected reward signal (Schultz, 2002; Tobler et al., 2005). However, Glu inputs to VTA in the β2-nAChRs KO mice likely remain intact and experience only minor changes in their activity as they are mostly modulated by the α7-containing nAChRs (Koukouli and Maskos, 2015). Further, re-expression of nAChRs within VTA restores high bursting levels. What is the mechanism that allows ACh to modulate bursting of the DA neurons? Our modeling shows that without β2-nAChRs, Glu excitation of VTA DA neurons remains in balance with inhibition mediated by neighboring VTA GABA neurons. Both neural groups receive partly overlapping Glu projections (Beier et al., 2015), and this may help to preserve the balance throughout different behavioral states. ACh inputs to VTA break the inhibition–excitation balance and, consequently, increase VTA DA neuron bursting. This makes the motivation and saliency signals carried by DA bursting vulnerable to the influence of nicotine and other substances affecting β2-nAChRs.

Second, a straightforward way to restore DA neuron activity does not work: re-expression of nAChRs on the DA neuron does not elevate its bursting and increases its firing rate only under the influence of nicotine. Why does direct excitation through β2-nAChRs not restore DA neuron activity? The answer may be in the similarity of the β2-nAChRs to other types of excitatory receptors that do not have voltage dependence: when driven by Glu inputs, DA neurons are most responsive to activation of their NMDA receptors (Overton and Clark, 1992; Chergui et al., 1993; Deister et al., 2009). By contrast, AMPA receptors are much less effective in eliciting bursts. The receptor difference that has been shown to be critical is the voltage dependence of the NMDA receptor: it is blocked by magnesium ions at lower voltages (Deister et al., 2009). This blockade allows the cell to repolarize and go into the next oscillation, whereas activation of the AMPA receptor does not allow for the repolarization and block oscillations altogether. Similar to AMPA receptors and distinct from NMDA receptors, β2-nAChRs do not have voltage dependence. Therefore, ACh input to DA neurons by itself is not effective in driving their bursting. This direct excitation can, however, significantly increase the DA neuron firing rate, which occurs under the influence of nicotine.

Third, ACh input and nicotinic application result in opposite changes in VTA DA neuron firing when β2-nAChRs are re-expressed only on VTA GABA neurons. In particular, the firing rate and especially the bursting of VTA DA neurons are sharply increased after the re-expression compared with the KO conditions. The application of nicotine, by contrast, decreases their firing rate and bursting. How can these modulations be reconciled? Our model uses the difference in the temporal pattern of receptor activation under the influence of ACh input versus nicotine: while ACh input produces pulsatile activation of the receptor, nicotine-induced activation is continuous and long lasting. A pulsatile input to the VTA GABA population can synchronize its firing and make its output to the DA neurons pulsatile as well. Again, such pulsatile inhibition of DA neurons may boost their bursting (Morozova et al., 2016a). Therefore, we predict that the opposite influence of ACh and nicotine through β2-nAChRs on GABA neurons is caused by the distinct temporal structure of the stimulation: pulsatile versus continuous.

In the experiments, the modulations of DA neuron firing introduced with the re-expression of β2-nAChRs on both DA and VTA GABA neurons closely reproduce that in WT mice under endogenous ACh inputs. However, these double re-expression experiments do not completely reproduce those in the WT mice during nicotine application. In particular, both firing rate and bursting evoked by nicotine are higher in the WT case (Figs. 2, 6). One possibility is that this difference is because of the altered expression of the receptors inside the VTA. It is plausible that the re-expression levels could be different in these conditions. However, the difference between the case of re-expressing the receptors in all VTA neurons and the WT occurs only during nicotine application. Therefore, we assume that this difference is because of the expression of β2-nAChRs outside of the VTA, which is not rescued by the re-expression. We assume that nicotine increases cortical activity in the WT mice more than in the β2 KO mice consistent with nicotine-induced activation of β2-nAChRs receptors on deep layer pyramidal neurons (Toyoda, 2018) and on the disinhibitory interneurons in the superficial cortical layers (Koukouli et al., 2017). We reproduced the elevated firing rate and bursting of DA neurons during nicotine application in the WT model with a modest increase in the average firing rate of Glu afferents to the VTA for the duration of nicotine application.

Note that we did not have to assume that the Glu input to the VTA changes its temporal structure under the manipulations performed experimentally. We propose a local VTA circuit mechanism for the changes in the DA cell activity. Previous modeling work has shown that the Glu afferent input that has a bursty temporal structure also increases the burstiness of the DA neuron (Morozova et al., 2016a). It is known that α7-containing nAChRs modulate Glu inputs to the VTA (Schilström et al., 1998, 2000, 2003) and impact DA neuron bursting in a subtle manner (Mameli-Engvall et al., 2006): β2-nAChRs appeared to exert an overwhelming control over all bursting under ACh and nicotine, while the α7-nAChRs manipulations in β2 intact animals affected bursting in a subpopulation of DA neurons with relatively low mean firing rates. These previous results suggest that α7-nAChRs have an impact on bursting, but in a manner that is strongly controlled by the β2-nAChRs. Previous data suggest that the deletion of α4 or α6 subunits does not cause significant changes in the firing and bursting of DA neurons (Exley et al., 2011). Therefore, different subtypes of nAChRs may control different sources of DA neuron bursting and, therefore, motivational and saliency signals associated with this bursting. However, in the experiments considered in the present study, the α7-nAChRs were not modulated. Hence, we must conclude that the changes we see in DA cell activity are because of β2-nAChR effects.

Previous results suggested that the coactivation of VTA DA and GABA neurons is required for the habit-forming effects of nicotine. How does the mechanism revealed in this article explain the requirement of GABA neuron activation? The coexcitation of DA and GABA neurons constitutes the conditions in which DA neuron bursting is maximized in the model. Indeed, excitation of DA neurons only can elevate their firing rate but does not group spikes into bursts. We predict that the additional excitation of the GABA neurons increases bursting in two ways. First, the tonic component of the inhibition mostly cancels the spikes without bursts rather than within bursts. This mechanism contributes most during nicotine application. Second, the excitation of GABA neurons by common inputs may also synchronize their firing and further promote bursting by providing pulsatile inhibition to DA neurons. This is a mechanism that provides background ACh-driven but not nicotine-driven DA neuron bursting because nicotine blunts the temporal profile of β2-nAChR activation and, thus, destroys synchronous pulses of GABA inhibition. Therefore, coactivation of DA and GABA neurons is required for DA neuron bursting and, thus, the motivation and saliency signals carried by these bursts, which are hijacked by nicotine and other addictive drugs.

## Synthesis

Reviewing Editor: Lorna Role, NINDS Decisions are customarily a result of the Reviewing Editor and the peer reviewers coming together and discussing their recommendations until a consensus is reached. When revisions are invited, a fact-based synthesis statement explaining their decision and outlining what is needed to prepare a revision will be listed below. The following reviewer(s) agreed to reveal their identity: Jerrel Yakel, Guillaume Drion.

The paper presents a computational model to explore the mechanisms by which endogenous acetylcholine and nicotine respectively affect VTA DA neuron firing rate and burstiness. It reproduces and compares different experimental conditions: “wild-type", systemic deletion of β2-nAChRs (i), (i) + recovery of β2-nAChRs in VTA DA neurons only, (i) + recovery of β2-nAChRs in VTA GABA neurons only, and (i) + recovery of β2-nAChRs in both VTA DA and GABA neurons.

The authors summarize experimental findings found in the literature and highlight prior contradictory effects of Ach and nicotine on VTA DA neurons firing in some conditions. The model was then used to uncover the mechanisms underlying VTA DA neuron response to ACh and nicotine and to explain the origin of apparently contradictory experimental results.

The authors show that the increase in firing rate of DA neurons in “wild type” conditions was due to the direct excitatory effect of cholinergic inputs onto DA neurons. The increase in DA burstiness was on the other hand due to an increase of GABA neuron activity and synchrony under cholinergic stimulation, which then promoted DA neuron bursting. The later effect required the activation of GABA neuron β2-nAChRs receptors to be phasic, which is not the case under nicotine application-hence explaining why nicotine application had the opposite effect than ACh in the “recovery of β2-nAChRs in VTA GABA neurons only” condition.

Overall the paper is well written, and the logic is clear. However, the reviewers have some questions and, in particular, are asking for a more thorough discussion of the following issues:

1. DA neuron “burstiness”.

The authors should discuss the role of bursting over spiking in VTA DA neurons and provide a more complete description of how bursting is quantified in practice. Specifically, include the methods used to compute the percentage of spikes in bursts in both experimental data and simulations. Are bursts mostly composed of “doublets” in your model, as Fig. 5 and 7 would suggest? Is it consistent with experimental results?

2. the potential role of receptor diversity in the VTA per se.

The authors should discuss and contrast both the role of receptor diversity within the VTA and the potential effects of systemic knock-out outside of VTA that could effect VTA DA and GABA neuronal activity, as well as how they would account for these phenomena in their model.

Specifically, the reviewers raise concerns with the authors suggestion that it is beta2 receptors outside the VTA that are the likely culprit in the observed effects. There is certainly receptor heterogeneity of the beta2-containing receptors within the VTA as well --; e.g. more than one alpha receptor subunit is expressed and there are likely more than one type of non-alpha7 receptor.

Likewise, global knock-outs can be complex. If the beta2 subunit could be knocked out locally; e.g. only in the VTA, might the model better match the nicotine data?

Additional comments and queries:

a. RE: Lines 57-61: “Deletion of the receptors on DA neurons eliminates their bursting mode (Mameli-Engvall et al., 2006), which is implicated in reinforcement (Bayer and Glimcher, 2005; Keiflin and Janak, 2015) (Fig. 1A). The role of β2-nAChRs on DA or GABA neurons in nicotine reinforcement was addressed using re-expression of the β2-nAChRs in specific cell populations of the VTA.”

The paper might gain clarity by properly defining what “reinforcement” and “nicotine reinforcement” mean in this context. Did the references mentioned show that the mechanism(s) implicated in “reinforcement” is the deletion of the receptors on DA neurons, DA neuron bursting, or both?

b. RE: Lines 68-70: “For the receptors re-expressed on the GABA neurons nicotine, in contrast to ACh, decreases the DA neuron firing rate and bursting (Fig. 2 blue).” please rephrase.

c. Lines 89-91: “There are other inputs to this circuit, e.g. from nucleus accumbense, but they are not affected by the KO manipulations in the experiments, and, thus cannot cause the differences.”

The KO of β2-nAChRs being systemic, it could indeed affect other inputs to the circuit. Do the authors mean that all other inputs lack β2-nAChRs? If not, this statement might not be correct. If yes, the authors should provide references.

d. Lines 269-272: “However, this required a modification of the nAChR conductances compared to the above re-expression cases (Table 2). The model shows that the ACh- and nicotine-induced modulations strongly depend on the balance between expression of these receptors on DA and GABA neurons.”

That would be interesting to have a more quantitative description of this dependency. Also, what would happen to the re-expression cases if one were to use these conductances instead of the previous ones?
