# Supplementary figures and images for: Distinct Temporal Structure of Nicotinic ACh Receptor Activation Determines Responses of VTA Neurons to Endogenous ACh and Nicotine
Source: eNeuro. 2020 Aug 21;7(4):ENEURO.0418-19.2020. doi: 10.1523/ENEURO.0418-19.2020 (PMC7470928; doi:10.1523/ENEURO.0418-19.2020)

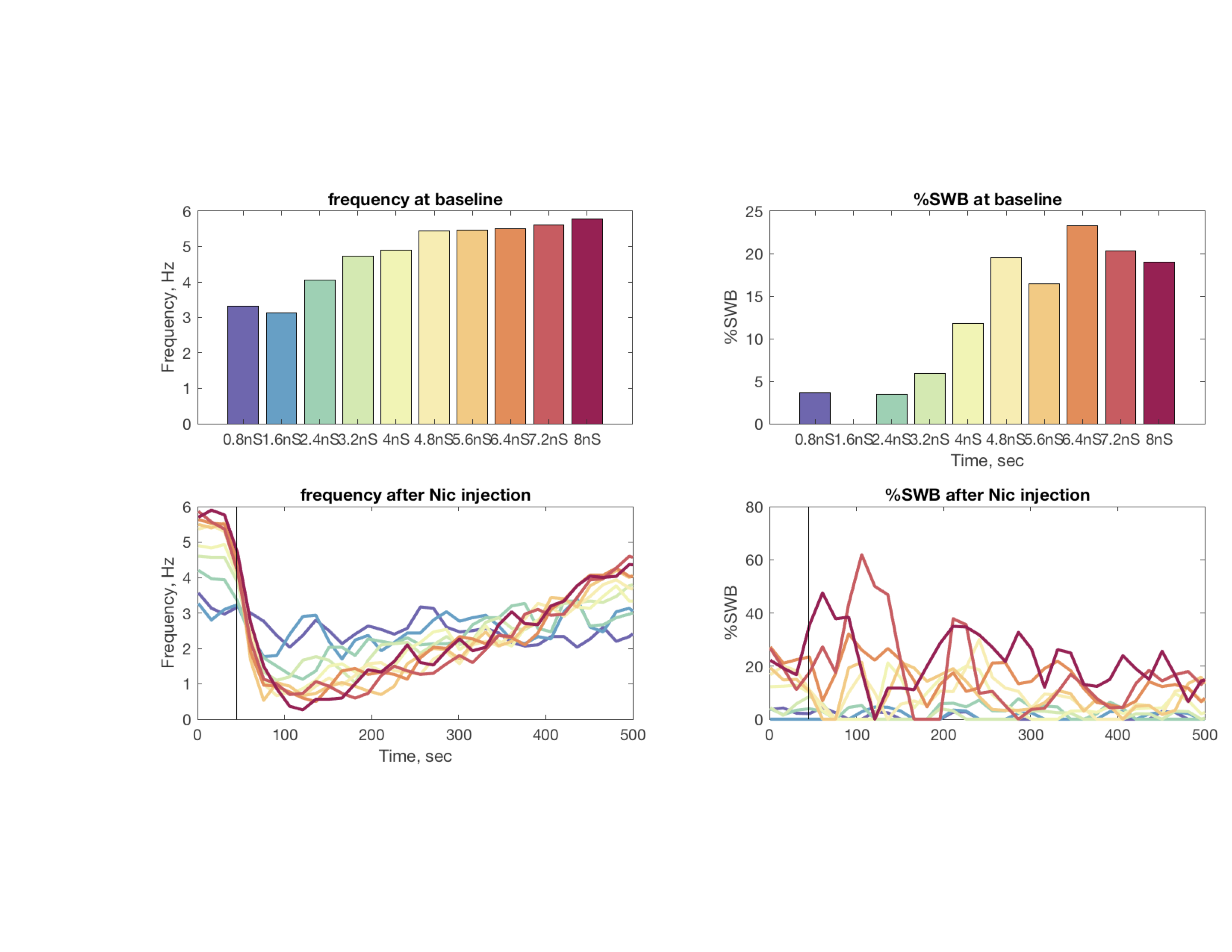

Supplement: Figure 6-1 — Parametric analysis of DA neuron responses to ACh and nicotinic inputs for different maximal conductances of nAChR current (mimicking different levels of expression of nAChRs) on GABA neurons. The range of low nAChR conductances on GABA neurons shows good correspondence with the experimental data. Download Figure 6-1, TIF file. [file enu-eN-NWR-0418-19-s02.tif]

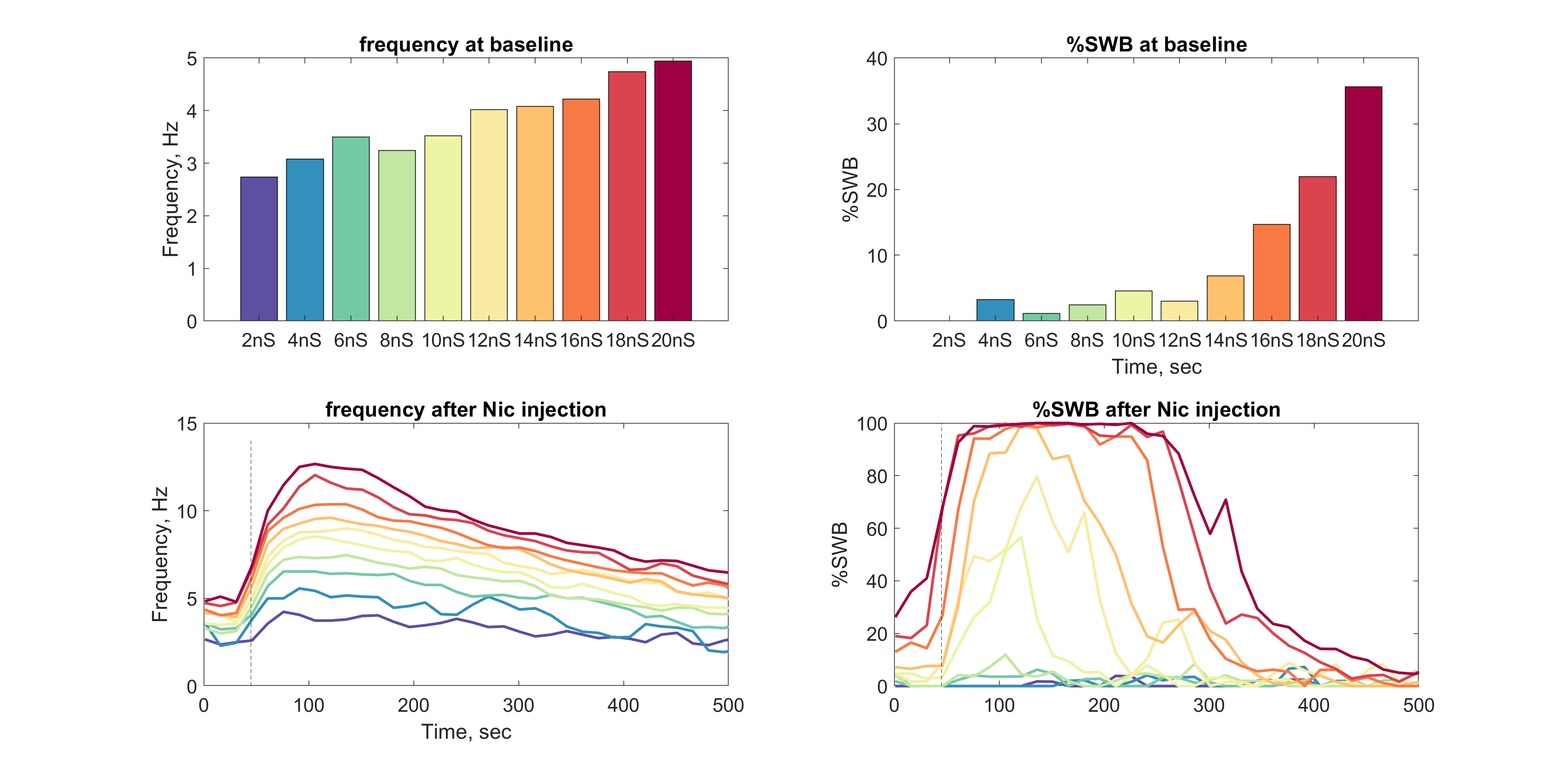

Supplement: Figure 5-1 — Parametric analysis of DA neuron responses to ACh and nicotinic inputs for different maximal conductances of nAChR current (mimicking different levels of expression of nAChRs) on DA neurons. The range of low nAChR conductances shows a good correspondence with the experimental data. Download Figure 5-1, TIF file. [file enu-eN-NWR-0418-19-s01.tif]
