# Supplementary material for: Distinct Temporal Structure of Nicotinic ACh Receptor Activation Determines Responses of VTA Neurons to Endogenous ACh and Nicotine
Source: eNeuro. 2020 Aug 21;7(4):ENEURO.0418-19.2020. doi: 10.1523/ENEURO.0418-19.2020 (PMC7470928; doi:10.1523/ENEURO.0418-19.2020)
Supplement: Table 1-1 — Model parameters. Download Table 1-1, DOCX file. [file enu-eN-NWR-0418-19-s04.docx]

Table 1-1: Model parameters

| **Parameter** | **Description** |  | **Value** |
| --- | --- | --- | --- |
| $c_{m}$ | Membrane capacitance | | $1\mu F/cm^{2}$ |
| $\overline{g}_{K}$ | Potassium conductance | | $1mS/cm^{2}$ |
| $\overline{g}_{Ca}$ | Calcium conductance | | $2.5mS/cm^{2}$ |
| $\overline{g}_{KCa}$ | Calcium-dependent potassium conductance | | $7.8mS/cm^{2}$ |
| $\overline{g}_{sNa}$ | Subthreshold sodium conductance | | $0.13mS/cm^{2}$ |
| $g_{leak}$ | Leak conductance | | $0.18mS/cm^{2}$ |
| $E_{K}$ | Potassium reversal potential | | $-90mV$ |
| $E_{Ca}$ | Calcium reversal potential | | $50mV$ |
| $E_{Na}$ | Sodium reversal potential | | $55mV$ |
| $E_{leak}$ | Leak reversal potential | | $-35mV$ |
| $E_{NMDA}$ | NMDA reversal potential | | $0mV$ |
| $E_{AMPA}$ | AMPA reversal potential | | $0mV$ |
| $E_{GABA}$ | GABA reversal potential | | $-90mV$ |
| $\tau_{aact}$ | AMPA receptor activation time | | $1ms$ |
| $\tau_{adeact}$ | AMPA receptor deactivation time | | $1.6ms$ |
| $\tau_{ades}$ | AMPA receptor desensitization time | | $6.1ms$ |
| $\tau_{adesrel}$ | AMPA receptor release from desensitization time | | $40ms$ |
| $\tau_{nact}$ | NMDA receptor activation time | | $7ms$ |
| $\tau_{ndeact}$ | NMDA receptor deactivation time | | $170ms$ |
| $\tau_{gact}$ | GABA receptor activation time | | $0.08ms$ |
| $\tau_{gdeact}$ | GABA receptor deactivation time | | $10ms$ |
